# Why do old flies die?

**DOI:** 10.18632/aging.100589

**Published:** 2013-08-10

**Authors:** Michael Rera, Rebecca I. Clark, David W. Walker

**Affiliations:** ^1^ Department of Integrative Biology and Physiology, University of California, Los Angeles, Los Angeles, CA 90095, USA; ^2^ Molecular Biology Institute, University of California, Los Angeles, Los Angeles, CA 90095, USA

As we get older we become more likely to get sick and, eventually, die. Although the underlying pathologies and major causes of death in elderly humans have been well documented, much less is known about the events leading to age-related death in the fruit fly *Drosophila melanogaster-* one of the premier model systems in aging research. What is the underlying pathology that limits the lifespan of a fly? Is it possible to predict when a fly will die based upon a loss of organ function? What accounts for the enormous variation in lifespan amongst individual flies within a population?

Recently, we have identified a physiological phenotype preceding death in *Drosophila* that allows us to identify, in any given population, individuals that will die in the next few days [1]. In this work, we show that all individuals show an altered control of intestinal permeability a few days prior to death *regardless of chronological age*. Interestingly, these same individuals also showed a striking increase in the expression of inflammatory markers (antimicrobial peptides, AMPs) as well as systemic metabolic defects, including impaired insulin/insulin-like growth factor signaling (IIS). Importantly, we observed that chronologically age-matched individuals, from the same population, without altered intestinal permeability do not show major changes in these parameters with age. This discovery suggests that, in *Drosophila* at least, these different phenotypes are tightly linked to one another and to the end of life. Indeed, we could independently identify flies that would die within a few days by selecting for increased AMP expression, and these flies showed systemic metabolic defects, including impaired IIS, and intestinal barrier failure. One interpretation of these findings, consistent with the ‘hyperfunction theory of aging’ [2], is that the overactivity of AMPs is driving pathology and directly leading to death. Alternatively, increased AMP expression may represent a benign marker of impending death, which may result, in large part, from other factors such as a loss of intestinal homeostasis and/or systemic metabolic dysfunction. As well as highlighting an important link between intestinal aging and organismal aging, this work may be telling us something about the very nature of the aging process itself. Our findings support a model where aging is composed of two consecutive phases, a first phase characterized by a growing likelihood of displaying intestinal barrier failure/inflammation/systemic metabolic dysfunction followed by a second phase leading to death. Remarkably, recent work from Cassandra Coburn, David Gems and collaborators has shown that intestinal cell death precedes organismal death in *C. elegans*, through a calcium-propagated necrotic wave [3]. Furthermore, a chronic state of inflammation [4] and the development of insulin resistance [5] are key hallmarks of human aging and have been linked to multiple age-onset diseases. Therefore, our findings, in *Drosophila*, may provide insight into the relationships between intestinal homeostasis, systemic aging and disease susceptibility in mammals.

The question of ‘why do old flies die?’ is not new. Seymour Benzer, one of the giants of 20^th^ century biology [6], used to ask this question to the members of his research group, on an almost weekly basis, during the 2000s. We haven't answered the question, of course. Our work does not reveal whether intestinal failure is the leading phenomenon provoking death in aged flies or only a symptom of an underlying disorder. Nor can we say why certain individual flies show intestinal failure at 10 days of age, whereas others can live for several months without losing intestinal barrier function. However, our ability to now identify those animals within a population that have entered the second phase of aging, and will die within the next few days, represents a major step towards answering these questions. And, ultimately toward defining the underlying causes of age-related cell, tissue and organ failure.
